# On the Vesicating Properties of the Potatoe Fly

**Published:** 1810-04

**Authors:** John Gorham


### *On the vesicating Properties of the Potatoe Fly.*

By Dr. JOHN GORHAM.

( Read before the Masachusetts' Society, February 3, 1808. )

IT is well known, that the only substance in common use among the physicians of this country, for the purpose of raising blisters on the skin is the cantharis, or meloe vesicatorius, a native of the countries bordering on the Mediterranean, and imported into the United States, often through the medium of Great Britain. The expence attendant on this mode of procuring it is undoubtedly great, and the possibility of obtaining, at all times, a constant supply, necessarily depends on the concurrence of numerous contingent circumstances, rarely within the control of the medical practitioner. Hence the physicians of America have acknowledged the importance of cultivating a more intimate acquaintance with those indigenous productions, which, from the activity of their preparations, and the easy modes of acquiring them for immediate use, are capable of being substituted for foreign medicines, and, consequently, of rendering them independent of those accidents and contingencies, whose occurrence, not many years since, was a source of embarrassment, and even of distress to the members of the medical profession on this side the atlantic. Among the small number of domestic medicines, whose active virtues have entitled them to a place in our materia medica, we may rank the Lytta Vittata, or potatoe fly, an insect, which, at certain seasons of the year, makes its appearance on the potatoe vine, and whose effects, when applied to the human system, are perfectly analogous to those of the Spanish cantharis.

The discovery of its peculiar properties was the result of accident, and it was first employed in the practice of medicine, by Dr. Isaac Chapman, of Bucks-county, Pennsylvania, whose conclusions, drawn from a partial investigation of the subject, may be found in a paper published in the 2nd volume of the New-York Medical Repository. In this, confining himself merely to a history of the effects, resulting from its external application, he is perfectly silent on its peculiar habits and modes of existence, and very limited in his description of its entomological characters. From this concise memoir we learn, that at the proper season of the year, it abounds in immense quantities on the potatoe plant, is easily procured and prepared for medicinal purposes, by the immersion of the vessel, in which which it is confined, in hot water, and its subsequent drying by exposure to heat; that the powder of this fly, when applied to the cuticle, produces irritation and the subsequent effusion of a serous fluid between that and the cutis vera, and, that in some cases slight strangury has supervened, in consequence of its partial absorption. The effects, resulting from its internal exhibition, have never been demonstrated by experiment; and he infers, only from analogy, that they are the same as follow the use of the cantharides. But though the properties of this valuable insect were thus made public, and its importance in our materia medica much enhanced by the facility with which it may be procured, it is probable that the demonstration of its activity was confined within the narrow sphere of Dr. Chapman's operations, since had it been introduced into general practice, and made an article of extensive commerce, its reputation, in the course of ten years, would, doubtless, have reached the physicians of this part of the country. Notwithstanding, however, that its existence and powerful effects have been known, for some time, to one or two medical practitioners in the interior of this State, it is only within a few days that these have been made public by its introduction into the materia medica of the pharmacopœia of the society by the title of Lytta Vittata,* or potatoe fly. It has, for some years past, been used as a vesicatory by Dr. Israel Allen, of Stirling, and it is to this gentleman that I am indebted for my knowledge of its existence there, and for a quantity sufficient to establish its character by extensive experiment.

The natural history of this insect is still very imperfect, and the following is all the knowledge we have been able to obtain from the publication of Dr. Chapman, and the verbal information of Dr. Allen. The Pennsylvanian fly "has a very near resemblance, in outward form, to the meloe (vesicatorius) alatus viridissimus nitens, antennis nigris of Linnæus, or Spanish flies, as they are commonly called, but is rather smaller than those brought from Spain, and of a very different colour; the head is of a very light red, with black antennæ; the elytra or wing cases are black, margined with pale yellow, and a stripe of the same colour extends along the middle of each of them; the tarsi have five articulations, the mouth is armed with jaws, and

* Dr. Woodhouse, of Philadelphia, has discovered other species of the Lytta.—Med. Rep. vol. 3.

and furnished with tarsi." "I found them," says Dr. Chapman, "in greatest number in potatoe patches; and when the potatoes are young, they frequently devour all the green leaves; they are frequently found among beets and garden purslane, of the leaves of which they are very fond." *

The fly of Massachusetts, at least in its dried state, is from four to six lines in length; its head and elytra are uniformly black, and the latter want the margin and stripe of yellow, observable in the insect of Pennsylvania. Its belly is ash-coloured, and in the cavity of the abdomen, as remarked by Dr. Chapman, "is a hard, white substance, about the size of a grain of wheat, which, when powdered, appears like meal, and when rubbed with water, forms a milk-like emulsion." The thickness of the potatoe fly, which is nearly uniform throughout, is from one quarter to one third its length. When soaked in water, its parts may be more accurately observed, and it will be found nearly to resemble some species of the beetle, by the thickness and hardness of the coverings which protect the membranaceous wings. When recently pulverised, it exhales a faint odour, which by some, has been compared with that of the powder of liquorice root, and by others, to althea ointment. It generally appears on the vines about the end of July and the first week in August, and in some years, according to the account of Dr. Allen, in such abundance, that many pounds of them may be collected in a day. They inhabit the soil at the foot of the plant; they ascend in the morning and afternoon, but generally avoid the heat of the sun at noon. As they fly with great difficulty, they are easily caught; and are prepared for medicinal purposes, by shaking them from the plant into hot water, and afterwards drying them by the rays of the sun.

By the very liberal manner in which I have been supplied with these flies by Dr. Allen, I have been able to institute an extensive series of experiments, in different forms, on different persons; and they have never failed, even in a single instance, of producing all the immediate effects which I anticipated from their external application or internal exhibition. As it is unnecessary, however, to detail the greatest part of these, I shall confine myself to the few, which from peculiarity of constitution, or error of management,

* Medical Repository, vol. 2, p. 174.

management, are most capable of illustrating the position we have assumed of their efficacy.

Case I.—June, 1807. Samuel White, an invalid, about 45 years of age, has, for some time past, been subject to occasional attacks of hiccough. During one of these paroxysms, after the unsuccessful exhibition of various medicines, had a large plaster covered with the powder of Lytta Vittata applied over the region of the diaphragm. During the succeeding night, the patient was very restless, and by frequent change of posture, gradually moved the plaster over a great part of the abdomen; and when he awoke in the morning, found it resting on his thigh. The whole surface of the former was completely vesicated, and incipient blisters were perceptible on the latter. The hiccough was relieved, though not removed. In consequence of this extensive vesication, a strangury supervened, which was soon removed by opiates and mucilaginous drinks. The excoriated surfaces were dressed with the common resinous ointment, and soon healed.

Case II.—June 10. Mary Wareham complained of intense pain in the head, for which blistering was prescribed. A plaster of the potatoe fly was applied, and the next day the vesication was found very perfect, accompanied with constant irritation of the part. The head-ache in a few days subsided, but the inflammation of the cutis was very considerable, continued for some days, and was succeeded by the formation of a number of boils about the size of hazle-nuts, and of extreme sensibility. These, at length, suppurated, and were finally healed by the application of the ung. resinosum cum oxido hydrargyri rubro.

Case III.—January 12, 1808. Sally Sharp came into the Alms-house for rheumatic pains, extending from the hip-joint to the knee of the right leg. The pain in the last joint being most troublesome, a plaster covered with powder of *cantharides* was applied to its surface, and kept in that situation for twenty-four hours; she experienced no irritation on the part, and when the plaster was removed, there was no vesication perceptible: it was again spread with *cantharides* and applied as before; but no obvious effect resulted from its second application. The flies were undoubtedly in good order, since a great number of persons had been completely blistered by powder from the same parcel. A plaster of *potatoe fly* was then substituted, and the next day I found, with much satisfaction, that it had been followed by a complete vesication. The blister, to to use the homely phrase of the patient, had run half a pint, and for a time the pains were alleviated.

If necessary, I might bring forward a multitude of cases in support of the efficacy of the Lytta Vittata; but these, I think, are sufficient to establish the fact, that as a vesicatory, they are at least equal, if not superior to the cantharides usually employed for that purpose in this country. Since then they are capable, even when externally applied, of producing a very considerable irritation in the urinary organs, we should judge, à priori, that a much more positive effect would result from their internal exhibition; and accordingly, I endeavoured to ascertain the truth of the position by experiment. Having about one drachm of the flies remaining of the parcel which I first received from Dr. Allen, it was infused in about six ounces of proof spirit, and the whole was undisturbed for some weeks. The tincture, therefore, I presume, was saturated, as the proportion of the flies was greater, and the infusion longer continued than the European Pharmacopœias have ordered in the preparation of the ordinary tincture of cantharides.

Case IV.—July 1807. Sarah Peirce, aged 70, was labouring under a paralysis of the neck of the bladder. Her urine passed off insensibly, and her situation rendered her uncomfortable both to herself and to others. The tincture of lytta vittata was prescribed in doses of ten drops twice a day, and these were gradually augmented to twenty drops three times in the twenty-four hours. In the course of few days a slight irritation was produced in the urethra, and the power of retaining her water had gradually increased. At the end of three weeks the incontinence of urine had altogether ceased.

Case V.— J. Nelson came under my care January 25, 1808, for a gonorrhœa of long standing. His symptoms were slight irritation during the passage of urine, and a discharge of a yellowish white matter, opake, and in considerable quantity. He was ordered to take fifteen drops of the tincture three times a day. On the 27th, the discharge of purulent matter had increased, accompanied with much scaldiug immediately after the passage of the urine; the dose was increased to eighteen drops three times a day. January 30th. Discharge of matter from the urethra lessened; complains of pain on making water, and of a soreness of the fauces, extending into the œsophagus. February 2. Discharge much abated, and the irritation ritation in the urethra rather less. In consequence, however, of the increased soreness which was now experienced even in the stomach, the medicine was discontinued. During its exhibition, the renal secretion was much greater than usual.

The tincture has been administered internally in many cases of diminished sensibility of the urinary organs, in gleets, and as a diuretic in dropsy; and it has uniformly been found in all to increase the discharge of urine, and to produce considerable degrees of irritation in the urethra and at the neck of the bladder.

It has also been applied externally as a vesicatory, and was always found to answer the purpose intended, though its effects were I think less complete than resulted from the application of the fly in substance.

From all therefore that I have been able to collect from the communication of Dr. Chapman, the verbal information of Dr. Allen, and the results of my own experiments continued for some months and made on more than fifty different cases, I think I shall be justified in drawing the following conclusions.

1. That the powder of the Lytta Vittata, or potatoe fly, when suffered to rest for some time on the cuticle, is capable of exciting inflammation and producing an effusion of a serous fluid between it and the cutis vera.

2. That the discharge in all cases is equal, and in many superior to that which results from the application of the powder of the cantharis; a remark which coincides precisely with the observations of Drs. Allen and Chapman. The superiority of the potatoe fly in this respect is doubtless owing, in some degree, to recent preparation. The cantharides are brought from a great distance, and there is sufficient reason for believing that their active qualities, like those of other medicines, in general, are impaired by age. Hence therefore this fact may be used as an argument in favour of the general adoption of our indigenous production, since it exists among us, and may be always obtained in a comparatively recent state.

3. That the saturated tincture of this fly possesses the vesicating property of the substance, but in rather an inferior degree.

4. That both the powder and the tincture are capable of inducing strangury by their external application.

5. That the vesication by the potatoe fly is effected in a shorter time than by the cantharis.

6. That

6. That when taken internally these flies produce all the effects, apparently in the same degree, as result from the exhibition of cantharides.

7. That the inflammation and subsequent secretion of purulent matter, are in general greater from the use of the potatoe fly than of the cantharis.

